# Genomic Selection with Allele Dosage in *Panicum maximum* Jacq.

**DOI:** 10.1534/g3.118.200986

**Published:** 2019-06-06

**Authors:** Letícia A. de C. Lara, Mateus F. Santos, Liana Jank, Lucimara Chiari, Mariane de M. Vilela, Rodrigo R. Amadeu, Jhonathan P. R. dos Santos, Guilherme da S. Pereira, Zhao-Bang Zeng, Antonio Augusto F. Garcia

**Affiliations:** * Luiz de Queiroz College of Agriculture / University of São Paulo (ESALQ/USP), Piracicaba, SP, Brazil,; † Embrapa Beef Cattle, Campo Grande, MS, Brazil, and; ‡ North Carolina State University (NCSU), Raleigh, NC

**Keywords:** Plant Breeding, Guinea Grass, Quantitative Genotyping, Polyploidy, Genotyping-by-sequencing (GBS), Recurrent Genomic Selection, Genomic Prediction, GenPred, Shared Data Resources

## Abstract

Genomic selection is an efficient approach to get shorter breeding cycles in recurrent selection programs and greater genetic gains with selection of superior individuals. Despite advances in genotyping techniques, genetic studies for polyploid species have been limited to a rough approximation of studies in diploid species. The major challenge is to distinguish the different types of heterozygotes present in polyploid populations. In this work, we evaluated different genomic prediction models applied to a recurrent selection population of 530 genotypes of *Panicum maximum*, an autotetraploid forage grass. We also investigated the effect of the allele dosage in the prediction, *i.e.*, considering tetraploid (GS-TD) or diploid (GS-DD) allele dosage. A longitudinal linear mixed model was fitted for each one of the six phenotypic traits, considering different covariance matrices for genetic and residual effects. A total of 41,424 genotyping-by-sequencing markers were obtained using 96-plex and *Pst*1 restriction enzyme, and quantitative genotype calling was performed. Six predictive models were generalized to tetraploid species and predictive ability was estimated by a replicated fivefold cross-validation process. GS-TD and GS-DD models were performed considering 1,223 informative markers. Overall, GS-TD data yielded higher predictive abilities than with GS-DD data. However, different predictive models had similar predictive ability performance. In this work, we provide bioinformatic and modeling guidelines to consider tetraploid dosage and observed that genomic selection may lead to additional gains in recurrent selection program of *P. maximum*.

Many agricultural and forage crops of economic importance are autotetraploids, such as potato (*Solanum tuberosum*) (Allard 1960), alfalfa (*Medicago sativa*) (McCoy and Bingham 1988), and guinea grass (*Panicum maximum* Jacq.) (Warmke 1954; Jank *et al.* 2014). For these species, marker alleles can be represented with different dosages ranging from 0 to 4. This allele dosage refers to the number of copies of the reference allele, ranging from *aaaa* for nulliplex, *Aaaa* for simplex (single dose), and so on up to *AAAA* (quadruplex), where *A* is the reference allele, for a biallelic marker, such as Single Nucleotide Polymorphisms (SNPs).

Historically, genetic analysis in polyploids has been based on single dose markers, mostly for building genetic maps (Wu *et al.* 1992; Hackett and Luo 2003). With the development of Next-Generation Sequencing (NGS) technologies and the advance of genetic and statistical methods, new possibilities have arisen to enable studies of the complex polyploid genomes for many crops (Garcia *et al.* 2013). In these species, the evaluation of SNP throughout the genome allows one to assess the relative abundance of each allele (Voorrips *et al.* 2011; Serang *et al.* 2012; Garcia *et al.* 2013; Hackett *et al.* 2013; Mollinari and Serang 2013; Schmitz Carley *et al.* 2017; Gerard *et al.* 2018).

According to Osborn *et al.* (2003), the allele dosage is expected to be related to different expression levels of some target traits. Thus, polyploidy can increase the potential variation in its genic expression, reflecting in phenotypic variation. Therefore, the inclusion of allele dosage information has become important for genetic studies in polyploid species (Garcia *et al.* 2013; Hackett *et al.* 2013; Endelman *et al.* 2018). In addition, it will allow the development of new statistical models in autopolyploid species, such as in the forage *P. maximum*.

*Panicum maximum* Jacq. (Syn. *Megathyrsus maximum* Jacq. B. K. Simon & S. W. L. Jacobs) is a perennial tropical autotetraploid forage grass (2n=4x=32) that reproduces by facultative apomixis (Warmke 1954), which means a high percentage of asexual reproduction by seeds. Cross-breeding started with the doubling of chromosomes by colchicine of rare diploid sexual plants (2n=2x=16) found in the center of origin in East Africa (Pernès 1975). This enabled the successful crossing between apomictic and sexual plants on a tetraploid level, giving rise to a progeny of sexual and apomictic hybrids in a 1:1 ratio (Savidan 1978). This means that it is possible to fix the hybrid vigor by apomixis in the $F_{1}$ generation. Also, superior sexual hybrids can be used as new parents in sexual × apomictic crosses or as donors of favorable alleles in sexual populations.

Few breeding generations separate the founder diploid accessions from the current sexual parents, and increase in the frequency of favorable alleles by selection for traits such as disease resistance, regrowth, biomass and seasonal yield, and nutritive value is necessary. Currently, conventional methods such as half-sib recurrent selection (RS) scheme (Hallauer and Carena 2012) have been carried out in sexual populations to increase the general combining ability of sexual parents. However, RS is a long term program as each cycle requires from three to five years for developing, evaluating, selecting and recombining superior plants. The evaluation phase needs two years for phenotyping the target traits in different harvests and seasons (rainy and dry seasons), and is the most time and expensive phase of the RS program. Thus, methods that accelerate the RS cycles will speed up the development of superior sexual parents for crossing with apomictic plants and increase the probability of releasing improved apomictic cultivars in the market. This is one of the goals of EMBRAPA, the Brazilian Agricultural Research Corporation. The unit Embrapa Beef Cattle is responsible for forage breeding programs, where the main crops are *Brachiaria* spp. and *P. maximum* (Jank *et al.* 2011). This research was established into the *P. maximum* recurrent genomic selection program.

Initially proposed by Meuwissen *et al.* (2001), genomic selection (GS) is an approach that uses statistical methods to predict breeding values from markers distributed throughout the genome. It uses all markers simultaneously to calculate the genomic estimated breeding values (GEBVs) of individuals for ranking and progeny selection (Meuwissen *et al.* 2001; Bernardo 2014). GS has recently been proposed as a useful tool for rapid genetic gains in RS (Massman *et al.* 2013; Müller *et al.* 2017; Zhang *et al.* 2017). It is a promising approach to accelerate cycles of recurrent selection since an established prediction equation can be used repeatedly for multiple cycles of selection (Müller *et al.* 2017). Furthermore, GS promises to increase genetic gain per unit of time, reduce costs for phenotyping, and increase the accuracy of selection.

Most of the available GS models were developed for diploids, and are not well adapted and evaluated for polyploid species. The huge challenge in working with polyploid species is in the correct distinction among different types of heterozygotes. Hidden heterozygotes lead to pseudo-diploid models, that are commonly applied in polyploid species (Annicchiarico *et al.* 2015; Biazzi *et al.* 2017). However, the impact of this approximation for GS models has only been reported in a few studies (Endelman *et al.* 2018; Oliveira *et al.* 2019).

The goal of this research was to develop and to evaluate predictive models for genomic selection in *P. maximum*. For this purpose, we compared different predictive models considering tetraploid and diploid allele dosage to obtain the GEBVs. The genotype calling was performed to allow the identification of different dose levels, showing all heterozygous genotypes and six different GS models were extended to incorporate the tetraploid allele dosage. To our knowledge, this is the first study that includes tetraploid dosage in whole-genome regression models for genomic selection in this tropical forage grass, combined with a high throughput genotyping in a sexual recurrent selection population of *P. maximum*.

## Materials and Methods

### Panicum maximum population

We generated *P. maximum* multi-parental population using 20 selected plants (JA, S7, S13, S16, A42, B87, T103, T4610, A47, A72, B107, C48, C16, B22, Y34, C54, B74, B96, BX4, and B103) as male parents and 19 sexual plants (the same set of plants except B107) as female parents in a polycross mating design. These parents were selected based on relevant agronomic performance in the forage breeding program at Embrapa Beef Cattle during the last 30 years. After the crossing process, we synthesized 19 half-sibs progeny families, each family composed of 30 individuals, resulting in a total of 570 tetraploid sexual plants.

### Phenotypic evaluation

The multi-parental population was evaluated in a split-plot randomized complete block design with six replications at Embrapa Beef Cattle, in Campo Grande city, Mato Grosso do Sul state, Brazil (20${}^{\circ }$27’S, 54${}^{\circ }$37’W, 530m). Half-sib progenies (represented by each female parent) were treated as the whole plot factor, while individuals were treated as sub-plot factors. Experimental units were 5 *m* by 2 *m* sized plots containing 5 individual plants of a family or 5 clones of one of three apomictic cultivars (B107, Mombaça, and Tanzânia). Thus, each block consisted of a total of 22 (19 half-sib families and three check cultivars) and a total of 110 individual plants (Figure S1). Therefore 660 plants were evaluated in the experiment, in which 570 were individuals from the 19 half-sib progenies and 90 were clones from the apomictic cultivars.

Each individual plant was evaluated for six traits: leaf dry matter (LDM - g/plant), regrowth capacity (RC), percentage of leaf blade (PLB - %), organic matter (OM - %), crude protein (CP - %), and *in vitro* digestibility of organic matter (IVD - %). The RC trait was evaluated seven days after the harvest using density notes and speed notes of sprouted stems. We considered these traits due their agronomic importance for forage breeding, especially for better forage quality properties for animal consumption. Thus, forage value is measured indirectly by being converted into animal products (such as meat, milk, leather, and furs) (Jank *et al.* 2011). The first three traits were evaluated for eight harvests, during the years of 2013 (four harvests), 2014 (one harvest), and 2015 (three harvests), and the last three were evaluated for four harvests, during the years of 2013 and 2015 (two harvests each).

### Statistical analysis of phenotypic data

For each trait, we fitted the following longitudinal linear mixed model to obtain predicted means later used in genomic selection analysis. The model is

$$y_{ijkl}=\mu +h_{l}+b_{k\left(l\right)}+p_{j\left(l\right)}+bp_{kj\left(l\right)}+t_{il}+e_{ijkl}$$ (1)

where $y_{ijkl}$ was the phenotypic value of the $i^{\text{th}}$ plant from the parent *j* in the block *k* at the harvest *l*, *μ* was the fixed overall mean, $h_{l}$ was the fixed effect of the $l^{\text{th}}$ harvest (l=1,…,L, with L=8 for LDM, RC, and PLB traits, and L=4 for OM, CP, and IVD traits), $b_{k\left(l\right)}$ was the random effect of the $k^{\text{th}}$ block (k=1,…,K, with K=6) at the harvest *l*, $p_{j\left(l\right)}$ was the random effect of the $j^{\text{th}}$ parent (j=1,…,J, with J=22) at the harvest *l*, $bp_{kj\left(l\right)}$ was the random interaction effect of block *k* and parent *j* at the harvest *l*, $t_{il}$ was the effect of the $i^{\text{th}}$ plant (i=1,…,I, with I=573) at the harvest *l*, and $e_{ijkl}$ was the random environmental error. The plant effects ($t_{il}$) were separated into two groups, in which $g_{il}$ was the random effect of the $i^{\text{th}}$ individual genotype ($i=1,\ldots ,I_{g}$, with $I_{g}=570$) at the harvest *l*, and $c_{il}$ was the fixed effect of the $i^{\text{th}}$ apomictic cultivar ($i=I_{g}+1,\ldots ,I_{g}+I_{c}$, with $I_{c}=3$) at the harvest *l*. For genotypes, the vector $g=\left(g_{11},\ldots ,g_{I_{g}L}\right)\prime$ was assumed to follow a multivariate normal distribution with zero mean and genetic VCOV matrix $G=G_{L}⊗I_{I_{g}}$, *i.e.*, g∼N(0,G). For residuals, e∼N(0,R), where $e=\left(e_{1111},\ldots ,e_{IJKL}\right)\prime$ and $R=R_{L}⊗I_{I\cdot J\cdot K}$. For parents, $p_{j\left(kl\right)}∼N\left(0,\sigma_{p}^{2}\right)$ and for blocks, $b_{k\left(l\right)}∼N\left(0,\sigma_{b}^{2}\right)$.

The choice of the genetic ($G_{L}$) and residual ($R_{L}$) VCOV matrices was performed hierarchically as follows. First, we picked the model with the best fit for the VCOV structures of the genetic effects among nine different VCOV structures (Table 1). This was done assuming an identity matrix (ID) as the VCOV matrix of the residual effects. Then, this picked model was evaluated assuming nine different VCOV structures for residual effects. Finally, the model with the best fit for the VCOV structure of the residual effect was selected. The models’ goodness of fitness were computed with Akaike Information Criterion (AIC) (Akaike 1974) and Bayesian Information Criterion (BIC) (Schwarz 1978). These analyses were performed in the R package ASReml-R (Butler *et al.* 2009). The generalized heritability (broad-sense heritability) for each trait was calculated using the same model as before mentioned but considering $G_{L}$ matrix as CS and $R_{L}$ as ID, according to Cullis *et al.* (2006) as:

**Table 1:** Variance and covariance structures examined for genetic ($G_{L}$ matrix) and residual effects ($R_{L}$ matrix)

Model	$n_{PAR}$*^a^*	Description
ID	1	Identical variation
DIAG	*L*	Heterogeneous variation
CS	2	Compound symmetry with homogeneous variation
CS${}_{Het}$	*L* + 1	Compound symmetry with heterogeneous variation
AR1	2	First-order autoregressive model with homogeneous variation
AR1${}_{Het}$	*L* + 1	First-order autoregressive model with heterogeneous variation
Po	2	Power model with homogeneous variation
Po${}_{Het}$	*L* + 1	Power model with heterogeneous variation
US	*L*(*L*+1)/2	Unstructured model

a The number of parameters for the models, where *L* is the number of harvests.

$${\hat{H}}_{C}^{2}=1-\frac{PEV}{2\sigma_{G}^{2}}$$ (2)

where, PEV is the prediction error variance (average variance of individual plant comparisons) and $\sigma_{G}^{2}$ is the genetic variance.

### Molecular data

Due to losses of individuals in the field, we sequenced a total of 530 offspring. DNA of these 530 offspring were extracted using the DNeasy Plant kit (QIAGEN) and sequenced along with female parents each repeated twice. To provide a higher sequence depth, genotyping-by-sequencing (GBS) was conducted in NextSeq 500 platform for 96-plex *Pst*1 libraries and following the protocol from Genomic Diversity Facility, Cornell University (Elshire *et al.* 2011). Raw data were analyzed using Tassel-GBS pipeline (Glaubitz *et al.* 2014) modified to obtain the exact read counts for each SNP allele (Pereira *et al.* 2018). As this pipeline requires a reference genome and *P. maximum* does not have one, we aligned the GBS tags against six pseudo-references from other diploid and tetraploid forage genomes, and *P. maximum* transcriptomes: (i) *Panicum hallii* genome (v. 2.0, DOE-JGI; ∼554 Mb arranged in 9 chromosomes and 8,405 scaffolds; diploid forage); (ii) *Panicum virgatum* genome (v 1.0, DOE-JGI; ∼1,230 Mb arranged in total of 18 chromosomes, 9 chromosomes named as A and B each, and 220,646 contigs; allotetraploid forage); (iii) *Setaria italica* genome (v 2.2; ∼405.7 Mb arranged in 336 scaffolds; diploid forage) (Bennetzen *et al.* 2012); (iv) *Setaria viridis* genome (v 1.0, DOE-JGI; ∼394.9 Mb arranged in 9 chromosomes and 724 scaffolds; diploid forage); (v) transcriptome of *P. maximum* obtained by EMBRAPA institution (43,803 transcripts with mean length of 841.334 bases); and (vi) transcriptome of *P. maximum* obtained by UNICAMP institution (138,853 transcripts with mean length of 695.658 bases). All the genomes are available in Phytozome website (http://www.phytozome.jgi.doe.gov/) (Goodstein *et al.* 2011). The transcriptomes were provided directly by the mentioned institutions. The transcriptome obtained by UNICAMP was published by Toledo-Silva *et al.* (2013). The transcriptome obtained by EMBRAPA has not yet been published. Bowtie2 algorithm (Langmead and Salzberg 2012) was used to align tags against each reference with -D and -R parameters defined as 20 and 4, respectively, and with very-sensitive-local argument. Common reads that aligned to the different genomes were called as different SNP markers. Then, we performed a preliminary analysis evaluating the prediction accuracy of models containing all the markers against models containing only non-redundant markers in genomic prediction models. As predictive accuracies between them did not differ, we chose to use all the markers in the subsequent analyses.

In Tassel-GBS pipeline, the minimum minor allele frequency (mnMAF) considered was 1%. The counting information derived from the Tassel-GBS pipeline was used to estimate the tetraploid allele dosage for each loci using SuperMASSA (Serang *et al.* 2012; Pereira *et al.* 2018). This software is designed especially for SNP calling in polyploid species based on probabilistic graphical models. In SuperMASSA software, the minimum overall depth considered was 25 reads and the model used was “Generalized Population Model”. Markers were fitted and filtered to ploidy 4. Triallelic SNPs and markers with more than 5% of missing data were filtered out. Since the selection of markers with up to 5% of missing data are a very strict filter, it is expected to include few percentage of errors due to the imputation process. The imputation was made using random sampling considering the frequency of each dose within each marker. Redundancy among markers was analyzed and displayed using R package circlize (Gu *et al.* 2014). The name of the markers was formed by: reference genome plus chromosome number plus position of SNP in the chromosome.

As linkage disequilibrium (LD) can affect the prediction accuracy of GS, the LD was estimated using squared Pearson correlation, $r^{2}$ (Vos *et al.* 2017). Correlations were calculated on tetraploid dosage (0, 1, 2, 3, and 4) between marker pairs with up 1500 bp of distance for each reference genome. The average $r^{2}$ between adjacent markers were calculated and, subsequently, the pairwise correlations were pooled over all chromosomes for each reference genome. A principal component analysis (PCA) was performed on the genotype data to detect population structure and displayed using ggplot2 (Wickham 2009). The relatedness among individuals was assessed computing (i) a pedigree-based additive relationship matrix (Amadeu *et al.* 2016), and (ii) a marker-based genomic relationship matrix (VanRaden 2008) using the R package AGHmatrix (Amadeu *et al.* 2016).

### Genomic prediction models using tetraploid dosage

Here, we generalized well known GS models for diploid to tetraploid species using the information of tetraploid allele dosage. Our response variable was the marginal predicted values of individual plants across harvests (BLUPs considering all harvests simultaneously). We evaluated both bayesian and frequentist approaches: Bayesian Ridge Regression (BRR) (Whittaker *et al.* 2000; Meuwissen *et al.* 2001); Bayes A (BA) (Meuwissen *et al.* 2001); Bayes B (BB) (Meuwissen *et al.* 2001); Bayes C (BC) (Habier *et al.* 2011); Bayesian LASSO (BL) (Park and Casella 2008); and Genomic Best Linear Unbiased Predictor (GBLUP) (VanRaden 2008).

All bayesian models expanded for tetraploid allele dosage (TD models) share the same predictive multiple linear regression model (a complete description of these models can be seen at Pérez and de los Campos (2014) and de los Campos *et al.* (2013)),

$$y=1_{n}\mu +\mathrm{X\beta}+\varepsilon$$ (3)

where, y (n×1) is the *n* adjusted entry mean response vector (mean values across harvests obtained from the phenotypic analysis); $1_{n}$ is a vector of 1’s; *μ* is a scalar representing the population mean; X (n×p) is the tetraploid allele dosage incidence matrix of *p* marker loci coded as $x_{ij}\subset \left\{0,1,2,3,4\right\}$ according to the copy number of the reference allele for individual *i* at marker *j*; β (p×1) is the vector of (unknown) marker with tetraploid dosage genetic (TDG) effects; and ε (n×1) is the vector of residual effects, $\varepsilon ∼MVN\left(0_{n},I\sigma_{\varepsilon }^{2}\right)$.

Different assumptions of TDG effects were evaluated. BRR-TD model assumes that all marker loci share the same normal prior distribution, $\beta_{j}|\sigma_{\beta }^{2}∼N\left(0,\sigma_{\beta }^{2}\right)$, where the common genetic variance hyperparameter ($\sigma_{\beta }^{2}$) follows a scaled inverse chi-squared hyperprior distribution $\sigma_{\beta }^{2}|d.f._{\beta },S_{\beta }∼\chi^{-2}\left(d.f._{\beta },S_{\beta }\right)$, where $d.f._{\beta }$ is the number of degrees of freedom and $S_{\beta }$ is the scale parameter of the distribution.

BA-TD model is an extension of the above model, which assumes that each TDG prior effects follows specific normal densities, $\beta_{j}|\sigma_{\beta_{j}}^{2}∼N\left(0,\sigma_{\beta_{j}}^{2}\right)$. As before, each specific genetic variance hyperparameter ($\sigma_{\beta_{j}}^{2}$) follows a scaled inverse chi-squared distribution. Due to its property, we expect that BA-TD model tends to shrink TDG effects with different prior strength (desirable for highly parameterized models, p≫n), as opposed to the BRR-TD that assumes a common genetic variance hyperparameter. Genetically, these assumptions mean that the analyzed traits are controlled by many genes of small effects and few genes of large effects.

BB-TD model is an extension of BA-TD model, that takes into account the TDG effects prior as a mixture of two normal densities, $\beta_{j}|\delta ,\sigma_{\beta_{j}}^{2},\pi ∼\pi \;N\left(0,\delta \right)+\left(1-\pi \right)\;N\left(0,\sigma_{\beta_{j}}^{2}\right)$, where *δ* is the genetic variance hyperparameter assumed as a known infinitesimal small value. In this model, we can interpret the mixture proportion (*π*) as a known expectation of a Bernoulli random variable, that is, the expectation of which mixture component best describes the TDG effects (Dos Santos *et al.* 2016).

BC-TD model is a parsimonious variant of the BB-TD model, which considers that all TDG effects follows a common mixture of two normal distributions, $\beta_{j}|\delta ,\sigma_{\beta }^{2},\pi ∼\pi \;N\left(0,\delta \right)+\left(1-\pi \right)\;N\left(0,\sigma_{\beta }^{2}\right)$.

BL-TD model assumes that all markers follows specific normal priors, with genetic variance hyperprior given by the product $\sigma_{\varepsilon }^{2}\sigma_{\beta_{j}}^{2}$. However, the key difference of BL-TD is the assumption that one component of genetic variance hyperparameters follows exponential distributions, $\sigma_{\beta_{j}}^{2}|\lambda_{\beta }∼Exp\left(\lambda_{\beta }\right)$. The hyperparameter $\lambda_{\beta }$ measures the knowledge (precision) about the genetic variance hyperparameter. As the usual procedure for all the above models, we assume the residual genetic variance hyperparameter follows $\sigma_{\varepsilon }^{2}|d.f._{\varepsilon },S_{\varepsilon }∼\chi^{-2}\left(d.f._{\varepsilon },S_{\varepsilon }\right)$.

The frequentist model GBLUP-TD was:

$$y=1_{n}\mu +\mathrm{Zg}+\varepsilon$$ (4)

where y, $1_{n}$, *μ*, and $\varepsilon_{ij}$ are the same as previously defined; Z (n×n) is an identity indicence matrix, and g (n×1) is the vector mapping the individuals total dosage genetic effects (random effect). The GBLUP-TD model assumes that the random variable g follows a multivariate normal distribution, $g∼MVN\left(0_{n},K^{*}\sigma_{g}^{2}\right)$, where $\sigma_{g}^{2}$ represents the genetic variance of the population and $K^{*}=0.99K+0.01A$, where K is the genomic relationship matrix and A the additive relationship matrix (VanRaden 2008; Aguilar *et al.* 2010; Isik *et al.* 2017). This normalization is necessary to obtain a invertible genomic relationship matrix (Isik *et al.* 2017) and the use of such weight does not result in critical differences in the outputs (VanRaden 2008; Aguilar *et al.* 2010). The K matrix, for tetraploid organisms, can be expressed as $K=\frac{WW^{T}}{tr\left(WW^{T}\right)/n}$, were W is the centered marker score matrix. Each $k_{i\prime i}$ on K can be interpreted as a correlation between genotypes of different individuals (genomic relationship), and each $k_{ii}$ as the correlation of the genotypes of an individual with itself (inbreeding). K and A matrices were computated using R package AGHmatrix (Amadeu *et al.* 2016).

To fit all bayesian models, we used the R package BGLR (Pérez and de los Campos 2014), choosing the default package settings for all known hyperparameters. To obtain the posterior distribution of the unknown parameters and hyperparameters, we used the Gibbs sampler with 20,000 iterations; the first 2,000 cycles were discarded as burn in. The GBLUP-TD model was analyzed using the R package ASReml-R (Butler *et al.* 2009).

### Model evaluation

The best statistical model was selected for each of the phenotypic traits. Cross-validation with fivefolds has been repeated 100 times for bayesian approaches (computationally intensive) and 1,000 times for the frequentist approach, to obtain an asymptotic empirical distribution of the predictive ability. In each replication, the population was randomly split into five disjoint subsets of genotypes. Whereas one subset was used as validation population (20% or 106 individuals), the remaining four were combined as training population (80% or 424 individuals) to predicte the left-out genotypes in the first population. Subsequently, another subset was used as validation population and the left-out genotypes of this set were predicted. These steps were repeated until all five subsets were used as validation population once.

We calculated Pearson correlation between observed (y) and predicted ($\hat{y}$) adjusted entry means for the validation sets, considering, simultaneously, all five cross-validations of each replication. Predictive ability was calculated as the mean of these correlations. In addition, we also derived the empirical distribution of the predicted residual error sums of squares (PRESS), given by the sum of squares of the difference between the predicted and observed adjusted entry means. The narrow-sense heritability was calculated as:

$${\hat{h}}^{2}=\frac{\sigma_{A}^{2}}{\sigma_{P}^{2}}$$ (5)

where, $\sigma_{A}^{2}$ is the additive genetic variance and $\sigma_{P}^{2}$ is the phenotypic variance from GBLUP analysis. Therefore, this estimate corresponds to a genomic heritability, using the additive relationship matrix from molecular markers.

### Genomic prediction models using diploid dosage

We also performed a comparison between GS models using diploid and tetraploid allele dosage (GS-DD and GS-TD models, respectively). To do so, the three possible heterozygotes (*Aaaa*, *AAaa*, and *AAAa*) were coded as a single heterozygote (*Aa*), while the two tetraploid homozygotes (*AAAA* and *aaaa*) as diploid homozygotes (*AA* and *aa*). Molecular data were filtered to eliminate markers containing only doses 0 and 1. This step was necessary since the offspring were closely related among them and most of the genotypes in the full data were classified as *aaaa* or *Aaaa* (84.27% and 12.57%, respectively). Therefore, the molecular data set decreased from 41,424 to 1,223 markers. In these new data, tetraploid molecular matrix contains 1,223 markers coded as 0, 1, 2, 3, and 4, and diploid molecular matrix contains the same 1,223 markers coded as 0, 1, and 2. Usual GBLUP model with diploid dosage (GBLUP-DD) was evaluated using R package ASReml-R (Butler *et al.* 2009), considering the genomics relationship matrix as described by VanRaden (2008). Bayesian models with diploid dosage were evaluated using R package BGLR (Pérez and de los Campos 2014). The model evaluation was performed as previously described.

### Data Availability

Phenotypic and molecular data and R code files can be found at: <https://github.com/leticia-lara/PM_data>. GBS data have been submitted to the NCBI Sequence Read Archive with the BioProject ID: PR-JNA510446. Additional information can also be found in the supplementary material. Figure S1 contains an illustrative scheme of the experimental design. Figure S2 contains the proportion of missing data in molecular dataset. Figure S3 contains the heatmaps of relationship matrices. Figure S4 contains the principal component analysis. Figure S5 contains the linkage disequilibrium analysis. Table S1 contains model selection for phenotypic traits. Table S2 contains the alignment rate for each reference genome. Table S3 contains the PRESS of predictive models for each trait. Table S4 contains the mean predictive ability of genomic selection models using tetraploid dosage for all markers. Table S5 contains the comparison between individual plant values based on half-sib family means and the genomic prediction of individuals. Supplemental material available at FigShare: https://doi.org/10.25387/g3.7762958.

## Results

### Phenotypic models

All VCOV structures selected for $G_{L}$ and $R_{L}$ matrices allowed for heterogeneous variation and/or correlations across harvests for genetic and residual effects (Table S1). When the selection by AIC was not in agreement with the selection by BIC, we calculated the differences between these two selected models considering both criteria and selected by the criteria that obtained the higher difference. For example, considering the OM trait and $G_{L}$ matrix, the Po*_Het_* had the lowest AIC value (with AIC criteria of 2774.400 and BIC criteria of 2826.809) and the Po had the lowest BIC value (with AIC criteria of 2782.262 and BIC criteria of 2817.201). The differences between these two selected models were 7.862 for AIC (2782.262 - 2774.400) and 9.608 for BIC (2826.809 - 2817.201). As BIC criteria had the higher difference, this criteria was used and the Po matrix was selected for $G_{L}$ matrix in OM trait. The most commom selected matrix for genetic effects was AR1${}_{Het}$, which was selected for CP and IVD, and different VCOV matrices were selected for residual effects. Generalized heritabilities were 0.66, 0.42, 0.48, 0.89, 0.85, and 0.43 for OM, CP, IDV, LDM, RC, and PLB, respectively. On average, traits evaluated in four harvests had lower heritability than traits evaluated in eight harvests.

### Genotype calling

Approximately 485 million of reads per lane were obtained from short read sequencing, where 81.73% were good barcoded reads. The alignment of 6,596,939 read tags using *P. hallii*, *P. virgatum*, *S. italica*, *S. viridis*, and two transcriptomes of *P. maximum* (Table 2) showed that the overall alignment ranged from 19.05 to 24.24%. Although transcriptomes obtained by UNICAMP had the highest overall alignment rate, transcriptomes obtained by EMBRAPA had the highest unique alignment rate, followed by *S. viridis* and *S. italica* (Table S2). A total of 476,904 markers were obtained as the sum of the final number of markers for each reference genome (Table 2).

**Table 2:** Overall alignment rate and total number of markers obtained after Tassel-GBS pipeline, allele dosage in SuperMASSA software, and selection of up to 5% of missing data (Filter NA), for each reference genome

Reference Genome	Overall alignment rate	Tassel-GBS	SuperMASSA	Filter NA
*Panicum hallii*	19.05%	77,105	12,835	6,945
*Panicum virgatum*	22.66%	84,119	11,230	5,598
*Setaria italica*	22.04%	92,494	15,047	8,066
*Setaria viridis*	22.07%	92,591	15,271	8,118
Transcriptome (EMBRAPA)	20.11%	74,049	14,129	7,665
Transcriptome (UNICAMP)	24.24%	56,546	9,777	5,032
Total	–	476,904	78,289	41,424

Due to the nature of GBS technique, the sequencing coverage of different samples are random. It is possible that the same genomic region was not sequenced for all samples. Furthermore, sequences that have mutation in the restriction site of the enzyme are also not observed (Elshire *et al.* 2011). Therefore, a large amount of missing data are expected. The reference genomes of *P. hallii*, *P. virgatum*, *S. italica*, and *S. viridis* had around 5,000 markers with 87% missing data (Figure S2). Markers with a high proportion of missing values were eliminated in subsequent steps. Besides, most part of the selected markers had 0% missing data after the Tassel-GBS pipeline. *S. italica*, *S. viridis*, and transcriptome obtained by EMBRAPA had more than 15,000 markers with 0% missing data.

**Figure 1:**
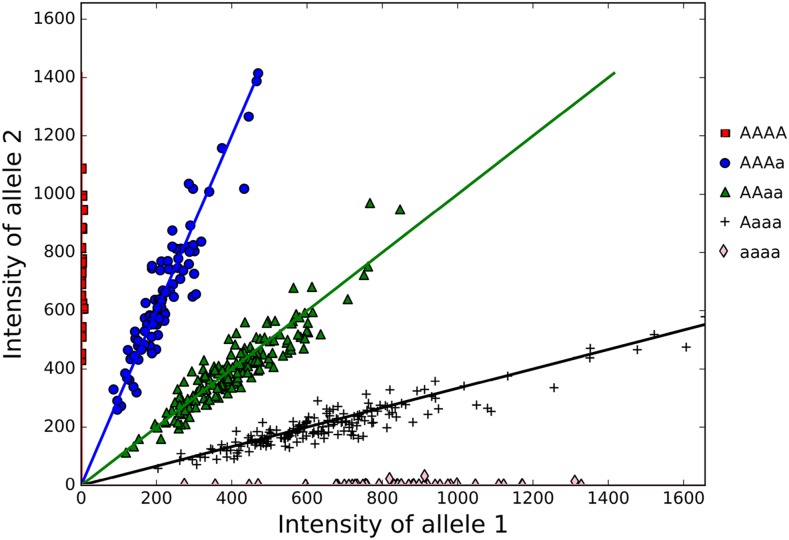
Tetraploid allele dosage for marker Hallii.1_3461595.

VCF files obtained from Tassel-GBS pipeline were used as input in the SuperMASSA software. A total of 78,289 markers was selected with minimum average depth for the population of 25 reads (Table 2). From this, 32,619 markers had more than 100 minimum overall depth. For example, the scatterplot of marker Hallii.1_3461595 (Figure 1) shows the intuition of how SuperMASSA uses count reads of two alleles to classify individuals according to their genotype using a probabilistic graphical model (Serang *et al.* 2012).

High level of missing data can impact the performance of GS models, and precise imputation of missing entries can be complex, especially when considering polyploid genomes. Therefore, markers were selected with up to 5% of missing data, aiming to reduce imputation bias. The final number of markers was 41,424, which were used in GS models (Table 2). Subsequently, imputation was made using random sampling and considering the dose frequency for each marker.

**Figure 2:**
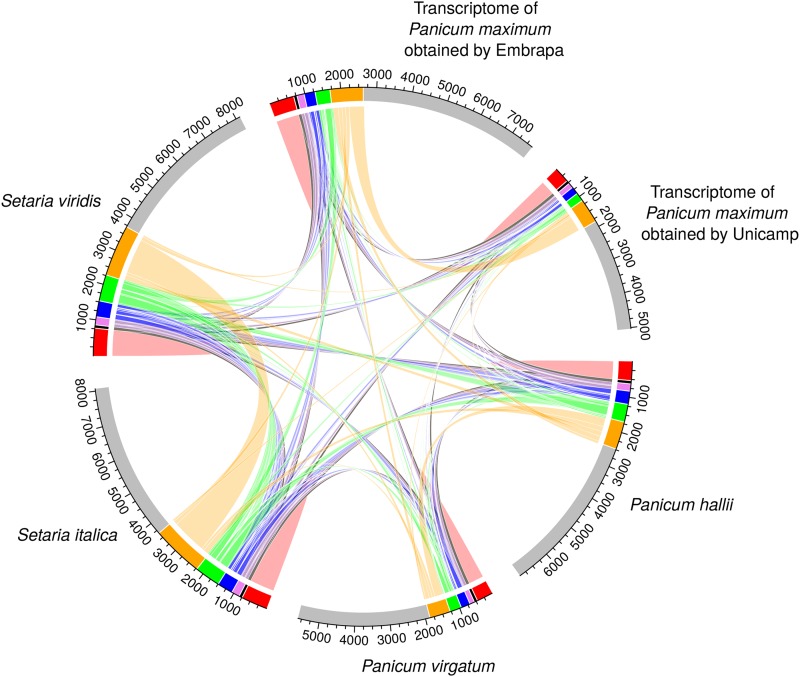
Redundancy among markers. Regions in red represent redundant markers within each reference, while regions in black, pink, blue, green, and orange represent redundant markers among six, five, four, three, and two references, respectively. Gray regions represent markers with unique information for each reference.

The redundancy among markers was inspected (Figure 2) and a large similarity was verified within three specific groups of reference: *Panicum* genus, *Setaria* genus, and transcriptomes. This result is expected due to phylogenetic proximity of the groups (Aliscioni *et al.* 2003; Bennetzen *et al.* 2012). More than half of the markers identified by the different reference genomes have non-redundant information, and 31,046 markers were classified as unique (74.95%). This may be due to the great genomic variability still persistent in each genome, since they are relatively new in terms of breeding.

### Population structure and linkage disequilibrium

A trace of population structure was detected for the additive relationship matrix based on pedigree information (Figure S3A) as expected, since these pedigrees consider only female parental information. However, no clear population structure was detected for the genomic relationship matrix based on molecular markers (Figure S3B). We also confirmed a relevant trace of population structure for female parents by principal component analysis of their genotype data, in which the three first principal components explained, respectively, 20.88, 11.98, and 10.23% of the total variability in these parents (Figure S4A). On the other hand, the principal component analysis for the 19 half-sib families (530 individuals) did not detected the population structure in these population. Their first three principal components explained, respectively, 6.20, 4.91, and 4.03% of the total variability in the breeding population (Figure S4B).

Average linkage disequilibrium (LD) between marker pairs was 0.3063, 0.3321, 0.3155, 0.3165, 0.2401, and 0.2810 for markers identified from *P. hallii*, *P. virgatum*, *S. italica*, *S. viridis*, and transcriptomes of *P. maximum* obtained by EMBRAPA and UNICAMP, respectively (Figure S5). The LD decay was lower for *P. hallii*, *S. italica*, and *S. viridis* than for others.

### Genomic prediction using tetraploid dosage

Genomic selection models were evaluated using all markers (41,424 markers) and only non-redundant ones (31,046 markers). The predictive accuracy did not differ between these two data sets (results not shown), since the predictive models deal well with multicollinearity. From now on, only predictive models using all markers will be presented.

**Figure 3:**
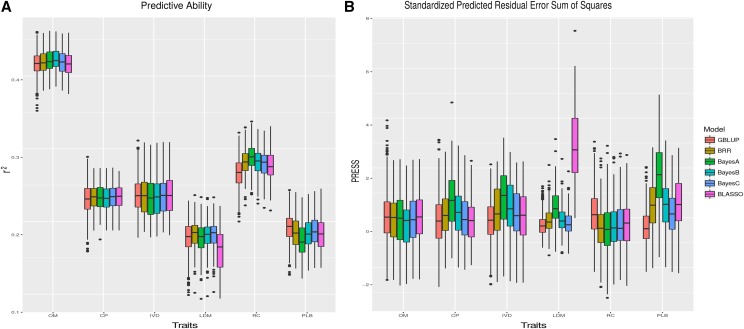
Comparison among six genomic selection models using tetraploid dosage (GS-TD) for organic matter (OM), *in vitro* digestibility of organic matter (IVD), crude protein (CP), leaf dry matter (LDM), regrowth capacity (RC), and percentage of leaf blade (PLB). Molecular data contains 41,424 markers. (A) Predictive ability. (B) Standardized Predicted Residual Error Sum of Squares.

Mean values of predictive ability ranged from 0.1610 (BL-TD for LDM) to 0.4229 (BB-TD for OM) (Table S4). LDM showed the lowest accuracies for all analyzed GS-TD models and OM showed the highest ones (Figure 3). No clear difference in predictive ability was observed among models (Figure 3A). The BL-TD model for LDM showed the highest estimates of standardized PRESS (Figure 3B, Table S3).

### Comparison between GS-TD and GS-DD models

**Figure 4:**
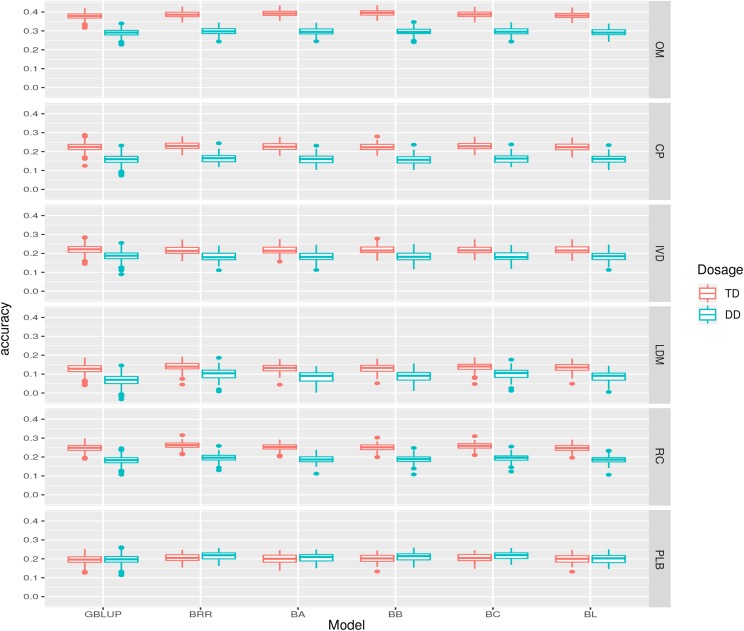
Comparison between genomic selection models using tetraploid dosage (GS-TD) and diploid dosage (GS-DD), for organic matter (OM), crude protein (CP), *in vitro* digestibility of organic matter (IVD), leaf dry matter (LDM), regrowth capacity (RC), and percentage of leaf blade (PLB). Molecular matrices containing 1,223 markers for each data set.

The mean predictive ability showed clear differences between GS-TD and GS-DD models (Figure 4 and Table 3). Almost all GS-TD models were superior to GS-DD models for most of the analyzed traits. The higest superiority was for LDM, with an average superiority of 50.96% for GS-TD model in relation to GS-DD model. The narrow-sense heritability ranged from 0.11 for LDM to 0.53 to OM. These results were consistent with those obtained for the predictive abilities, which is expected since both were calculated considering the additive effects (Table 3).

**Table 3:** Mean predictive ability of genomic selection models using tetraploid dosage (GS-TD) and diploid dosage (GS-DD) for organic matter (OM), crude protein (CP), *in vitro* digestibility of organic matter (IVD), leaf dry matter (LDM), regrowth capacity (RC), and percentage of leaf blade (PLB). GS-TD and GS-DD models with the highest mean predictive ability for each trait are indicated in bold. Molecular matrices containing 1,223 markers for each data set. The broad-sense heritability is H^C2 and the narrow-sense heritability is h^2

Model	OM	CP	IVD	LDM	RC	PLB
GBLUP-TD	0.3782	0.2237	**0.2211**	0.1282	0.2475	0.1957
GBLUP-DD	0.2905	0.1578	0.1869	0.0685	0.1829	0.1968
BRR-TD	0.3866	**0.2299**	0.2157	**0.1390**	**0.2621**	0.2058
BRR-DD	0.2966	0.1633	0.1837	0.0997	0.1951	0.2155
BA-TD	0.3931	0.2251	0.2174	0.1308	0.2514	0.2001
BA-DD	0.2958	0.1585	0.1835	0.0854	0.1876	0.2067
BB-TD	**0.3955**	0.2242	0.2190	0.1311	0.2501	0.2004
BB-DD	0.2960	0.1560	0.1843	0.0886	0.1885	0.2110
BC-TD	0.3879	0.2282	0.2186	0.1374	0.2577	0.2049
BC-DD	0.2958	0.1612	0.1855	0.1006	0.1944	**0.2161**
BL-TD	0.3821	0.2233	0.2187	0.1333	0.2468	0.1991
BL-DD	0.2923	0.1594	0.1849	0.0870	0.1853	0.1998
Average GS-TD	0.3872	0.2257	0.2184	0.1333	0.2526	0.2010
Average GS-DD	0.2945	0.1594	0.1848	0.0883	0.1889	0.2076
Average	31.48	41.59	18.18	50.96	33.72	−3.18
superiority						
${\hat{H}}_{C}^{2}$	0.66	0.42	0.48	0.89	0.85	0.43
${\hat{h}}^{2}$	0.53	0.23	0.25	0.11	0.31	0.12

## Discussion

The aim of this study was to develop predictive models considering allele dosage for autotetraploid species, with applications in *P. maximum*. We extended six different GS models to autotetraploid species and compared the accuracy of predicted breeding values of these models. To the best of our knowledge, this is for the first time where the efficiency of genomic selection models considering the information from hidden heterozygotes in the autotetraploid tropical forage *P. maximum* has been reported. Furthermore, we evaluated strategies for modeling residual effects during phenotypic analysis, and performing quantitative genotype calling in autotetraploid species. This methodology can be applied to other autotetraploid species as well as can be extended to species with higher ploidy levels.

Before the development of GS models considering tetraploid allele dosage (GS-TD models), it is important to perform a phenotypic analysis carefully since the sucess of GS in breeding for quantitative traits largely depends of phenotyping process (Cabrera-Bosquet *et al.* 2012). We performed a two stage approach for genomic selection, where we considered only phenotypic data in the first stage and incorporated molecular data in the second stage. In the first stage, for each trait, we fitted a longitudinal linear mixed model and treated genotypes as random. We recognized there is a discussion about fit genotype as random in the first stage (Schulz-Streeck *et al.* 2013). However, as we have multiple harvests and we are modeling genotype effects nested within harvests, with VCOV structure for genotype values across harvests, the model requires to treat genotype as random. The gain in information by incorporating the correlations among harvests is corroborated in the VCOV structures selection, in which the matrices allowing heterogeneous genetic and residual variation as well as allowing correlation among harvests provided a better fit than other models for most analyzed traits (Table S1). Moreover, there was reported in the literature that the use of BLUPs (Best Linear Unbiased Predictor, and in this case, it refers to the marginal predicted values of individual plants across harvests) does not result in significant differences for selection purposes (Galli *et al.* 2018), and it has been already applied in other GS studies as a simple approach to include correlations among traits/harvests/environments (Asoro *et al.* 2011; Resende *et al.* 2017; Ferrão *et al.* 2017; de Oliveira *et al.* 2018). Furthermore, we calculated the deregressed BLUPs, but the GS models had lower predictive ability than using the marginal predicted values of individual plants across harvests (results not shown).

In the second stage, molecular markers were considered in the predictive models. For this, the genotype calling took into consideration the allele dosage, discriminating among the five possible genotypes. According to Uitdewilligen *et al.* (2013), a high sequence depth is required to identify the genotypic class accurately for autotetraploid species, where 60–80 depth leads to 98.4% accuracy in genotypic calls. Reads were also selected with minimum overall depth of 100 reads. However, as the predictive ability of GS-TD models was similar for both criteria (results not shown), we chose to keep only the analysis with an overall depth of 25 reads (approximately 78.7% of markers selected with minimum overall depth of 25 reads were also selected with minimum overall depth of 100 reads). This is a less strict filter for the practical pipeline of breeding and contains a larger number of markers.

The underlying assumption of genomic selection is the presence of SNPs at some loci in linkage disequilibrium (LD) with Quantitative Trait Loci (QTL) alleles that affect traits which are subject to selection (Calus *et al.* 2008). LD represents the non-random association between alleles at different loci and it can be estimated using the correlation among markers when the SNP alleles at those loci are coded with numerical values. Vos *et al.* (2017) calculated several estimators for LD in a simulated and real panel of tetraploid potato and concluded that LD${}_{1/2,90}$ values provides the most consistent estimates of LD decay. This estimator consists of 90% percentile $r^{2}$ the short-range LD. Short-range LD is calculated across a defined interval of physical distances between marker pairs (Vos *et al.* 2017). One major reason for the minor differences in prediction accuracies among prediction models is the high level of LD found in breeding populations (Riedelsheimer *et al.* 2012). Riedelsheimer *et al.* (2012) obtained similar accuracies of GS models in elite maize germplasm, which had high level of LD. Accuracies did not differ regardless whether the effect of large QTL were precisely captured or spread over a larger region. In this work, we also obtained a high level of LD between markers pairs with up 1500 bp of distance ($r^{2}$ from 0.2401 to 0.3321), which can explain the similarity among GS-TD models for prediction purposes.

Population structure is another important factor in GS and can result in biased estimates of the predict ability when it is not taken into account (Riedelsheimer *et al.* 2013). As our population is composed by half-sib families, a stratified sampling was performed for the cross-validation process, in which 20% of individuals in each family were taken from the validation population and 80% from the training population. Therefore, all families were represented in both populations. The GS models and the evaluation process were performed as described in Materials and Methods section. We did not observe differences in the predictive abilities between stratified sampling and random sampling (results not shown) and we are presenting in this paper only results using random sampling. This similarity is expected, since no clear population structure was detected in this population of *P. maximum* (Figure S3 and S4B). When we observe the genotypes of the parents, few parents are different from the others (Figure S4A). The absence of population structure occurs because most parents are closely related and the individuals analyzed have a high level of relationship among them. Similar results have been reported in wheat (Arruda *et al.* 2015) and in potato (Sverrisdóttir *et al.* 2017), with typical values for data with family structure, but without substantial population structure. As reported by Sverrisdóttir *et al.* (2017), the absence of population structure could in principle be caused by an extremely narrow genetic base of the parents. This is in line with our study since all parents were derived from a common female tetraploidized ancestor.

Synthetic populations are usually formed by crossing several parents and posteriorly cross-pollinating the F${}_{1}$ individuals for one or several generations (Falconer and Mackay 1996). These populations have played an important role in quantitative genetic research on gene action in complex heterotic traits and comparison of selection methods (Hallauer *et al.* 2010; Schopp *et al.* 2017). As in our population, the number of parents is usually small and parents are often related, leading to small effective size ($N_{e}$) (Müller *et al.* 2017). We estimated broad and narrow sense heritabilities for the evaluated traits. Besides we did not expect high heritabilities for traits related with yield, we observed a high broad sense heritability (0.89) and low narrow sense heritability (0.11) for LDM (Table 3). Such low value reflects the difficulty to breed for this trait. Differences in magnitude between these estimates were previously reported by Resende *et al.* (2014b), which observed broad-sense heritability ranging from 0.47 to 0.75 and narrow-sense heritability ranging from 0.15 to 0.24 for LDM considering different years of evaluation. For individual harvests, the same authors found a narrow-sense heritability of 0.05 for LDM. Similar to our results, broad-sense heritabilities were reported in *Paspalum* for LDM with 0.82 and PLB with 0.55 (Pereira *et al.* 2017). In respect to the nutritive traits, moderate values were reported by Matias *et al.* (2016) for narrow-sense heritability considering the mean of seven harvests in *Brachiaria decumbens* progenies for CP (0.31) and IVD (0.14). Similar moderate values for CP and IVD were also reported in our study (Table 3).

The prediction of the breeding values was made using the marginal predicted values of individual plants across harvests that were obtained in the first stage of the analysis. The goals were to select individuals in the present recurrent selection cycle (already phenotypically evaluated) as well as to select non-phenotyped individuals from the next cycle. Since one selection cycle requires three to five years to complete (Resende *et al.* 2014a), *P. maximum* breeding program with genomic selection will reduce from five to one year for each recurrent cycle. This one year is necessary to grow the selected plants and cross them to obtain the new population. In addition, the *P. maximum* breeding programs for releasing cultivars will be benefited since superior sexual plants can be selected every year to, posteriorly, cross with apomictic plants. From these crosses, new apomictic hybrid combinations will be obtained and tested as new cultivars for releasing the best one in the market. Lipka *et al.* (2014) applied genomic selection in *P. virgatum* species with the objective of evaluating genomic selection efficiency to accelerate breeding cycles in this species. Since *P. virgatum* is an allotetraploid species, the authors used diploid dosage and obtained high prediction accuracy for most of the traits, using association panel and considering seven morphological and thirteen biomass quality trais. Although analyzed traits were different from ours, the range of values were similar. The higher mean prediction accuracy obtained by Lipka *et al.* (2014) was 0.52 for standability and the lower was -0.08 for minerals. Our higher mean predictive ability was 0.4229 for organic matter and the lower was 0.1610 for leaf dry matter (Table S4). Similar accuracies were also obtained for oats (Asoro *et al.* 2011), maize (González-Camacho *et al.* 2012), and rice (Spindel *et al.* 2015). Despite the difference among traits, the accuracy did not differ among models. Besides that, we suggest to use GBLUP-TD model for all traits, since it is more intuitive for breeders and it is less computational demanding.

We also compared the predictions of individual plant values based on genomic prediction at the level of half-sib family means with that at the individual level. To achieve this, we removed the maternal effect of the phenotypic model and included it in the genomic selection model. Then, we predicted individuals based only on the maternal effects and compared with prediction of individuals (Table S5). The genomic predicted values are more accurate because they capture not only information on the family means, but also the within-family deviations. The accuracy of prediction using only maternal effect ranged from 0.0452 for PLB to 0.1843 for LDM. The prediction of individuals using molecular markers and family effect ranged from 0.1786 for LDM to 0.3466 for OM. Therefore, the GS can operate on individual plants without needing trait data on sibs with considerable accuracy of prediction and genetic gains with selection.

There are few studies on GS for autotetraploid species using allele dosage, most of them are for potato (Slater *et al.* 2016; Endelman *et al.* 2018; Stich and Inghelandt 2018). To our knowledge, this is the first study that applied genomic selection with allele dosage in an autotetraploid tropical forage grass. Slater *et al.* (2016) developed an extension of genomic relationship matrix proposed by Yang *et al.* (2010) for autotetraploids and applied genomic selection in potato. The authors found accuracies ranging from 0.2, under conditions of low heritability and small reference populations, to 0.8 in larger reference populations. Endelman *et al.* (2018) and Stich and Inghelandt (2018) analyzed the inclusion of additive and non-additive components (digenic dominance and two order epistasis) for the accuracy of predictive models. The inclusion of dominance effects in GS when selecting the per-se performance of the individuals is fundamental, mainly when working with species that are highly heterozygous, which allows dominance effects to contribute to phenotypic variation (Stich and Inghelandt 2018). Concordantly, Endelman *et al.* (2018) extended the genomic relationship matrix proposed by VanRaden (2008) for autotetraploids and highlighted the importance of genotypic and additive values for selection. However, for both studies, when the goals is selecting new parents for several cycles, only the additive value needs to be considered since the contribution of digenic dominance diminishes exponentially over the generations (Gallais 2003; Endelman *et al.* 2018), in other words, non-additive effects are less efficiently transmitted to progeny. In our work, we modeled only additive effects since our study aims to select superior sexual parents in a recurrent genomic selection program in the current and next selection cycles.

A comparison of GS-TD models with GS models considering the usual diploid allele dosage (GS-DD models) was also performed. Although being a rougly approximation, this strategy has been applied in several polyploid crops, such as alfafa (Annicchiarico *et al.* 2015; Biazzi *et al.* 2017), and sugarcane (Gouy *et al.* 2013), as GS software for polyploids have only recently emerged. To investigate the impact of using diploidized markers, Endelman *et al.* (2018) also compared diploid and tetraploid dosage. The authors observed that the accuracy of the diploidized model was consistently lower. In our study, the accuracy of GS-DD models were also lower than for GS-TD models for all traits (Table 3 and Figure 4). This reinforces that GS models using tetraploid allele dosage are superior for autotetraploid populations, and extensions can and should be applied to other ploidy levels.

### Implementation of genomic selection in forage breeding

Recent research shows the potential of genomic selection to reshape crop breeding programs. In particular, the results obtained here imply that GS has great potential for *P. maximum* breeding programs, especially in a recurrent genomic selection program.

**Figure 5:**
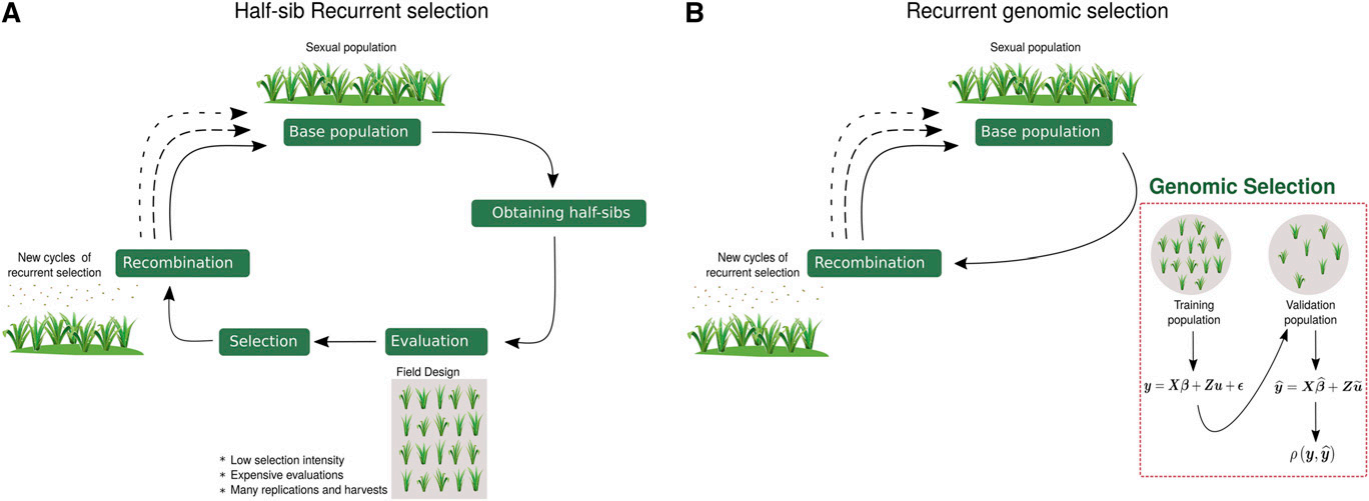
Forage breeding program in a: (A) recurrent phenotypic selection. (B) recurrent genomic selection.

Usually, one cycle of half-sib recurrent selection is split in: i) development of progenies; ii) phenotypic evaluation; and iii) selection and recombination of the best selected individuals to obtain an improved population (Figure 5A). The recurrent genomic selection is a modification to get shorter breeding cycles and greater genetic gains with selection. Therefore, genomic prediction is implemented by genotyping and phenotyping the base population and estimating marker effects to predict hybrid performance in the subsequent recurrent selection cycles and recombine the best individuals based only on the GEBVs (Figure 5B). The persistency of predictive accuracy in GS is fundamental for practical breeding because it determines the number of cycles that can be advanced until it is necessary to retrain the predictive models (Müller *et al.* 2017). This is because in each cycle of recombination and selection, the individuals of breeding population can accumulate genetic diversity and gene frequencies may differ from the training population (Heffner *et al.* 2010; Bassi *et al.* 2016). Therefore the breeder needs to update the GS models by phenotyping the population and re-estimating marker effects each three recurrent selection cycles (Heffner *et al.* 2009) or whenever necessary.

To compare the efficiency of GS approach with phenotypic breeding programs, the breeders’ equation can be used. In this equation, expected genetic gains per unit of time is defined as ${\text{Δ}}_{G}=\left(ir\sigma_{A}\right)/T$, where *i* is the selection intensity, *r* is the selection accuracy, $\sigma_{A}$ is the square root of the additive genetic variance, and *T* is the length of time to complete one breeding selection cycle (Falconer and Mackay 1996). The success of GS approach is determined by its ability to predict phenotypes that were not evaluated as well as by its ability to increase the rate of ${\text{Δ}}_{G}$ while maintaining affordable costs. Assuming similar selection intensity and similar genetic variance for both methods, greater gains per unit of time can be achieved as long as the reduction in the time of each breeding cycle by GS compensates for the reduction in selection accuracy (Bassi *et al.* 2016). Furthermore, GS becomes cheaper than phenotypic selection for traits that have a long generation time or are difficult and expensive to evaluate.

Another crucial aspect for implementation of genomic selection in forage breeding programs is the generation of accurate genotypic data. Several softwares have been developed to perform quantitative genotype calling for autotetraploid species, such as R packages fitTetra (Voorrips *et al.* 2011) and ClusterCall (Schmitz Carley *et al.* 2017); for “diploidizing” tetraploid species, such as R package breadarrayMSV (Gidskehaug *et al.* 2010); and species with any ploidy level, such as R packages fitPoly (Voorrips *et al.* 2011), updog (Gerard *et al.* 2018), and software SuperMASSA (Serang *et al.* 2012; Pereira *et al.* 2018). Therefore, the evaluation of allele dosage for polyploid species has become more accessible by several available softwares.

This is the first work of genomic selection in the tropical forage grass *P. maximum* which uses a high throughput genotyping and considers tetraploid allele dosage in Bayesian and frequentist models. GBS and allele dosage showed to be promising strategies for genomic analysis in autotetraploid species. Furthermore, the accuracy of predictive models and the time reduction in the breeding cycles justifies the implementation of genomic selection in *P. maximum* breeding programs.
